# Feeding the critically ill child in intensive care units: a descriptive qualitative study in two tertiary hospitals in Ghana

**DOI:** 10.1186/s12887-021-02854-2

**Published:** 2021-09-10

**Authors:** Alhassan Sibdow Abukari, Angela Kwartemaa Acheampong

**Affiliations:** School of Nursing, Wisconsin International University College-Ghana, P.O Box LG, Accra, Ghana

**Keywords:** Feeding, Enteral, Parenteral, Cup feeding, Critically ill child, Intensive care units, Ghana

## Abstract

**Background:**

Critically ill children require optimum feeding in the intensive care units for speedy recovery. Several factors determine their feeding and the feeding method to adopt to address this phenomenon. The aim of this study was to explore and describe the feeding criteria of critically ill children at the neonatal and paediatric intensive care units.

**Methods:**

A descriptive qualitative design was used to conduct the study. Six focus group discussions were conducted, and each group had five members. In addition, twelve one-on-one interviews were conducted in two public tertiary teaching hospitals in Ghana and analyzed by content analysis using MAXQDA Plus version 2020 qualitative software. Participants were selected purposively (*N* = 42).

**Results:**

The decision to feed a critically ill child in the ICU was largely determined by the child’s medical condition as well as the experts’ knowledge and skills to feed. It emerged from the data that cup feeding, enteral, parenteral, and breastfeeding were the feeding processes employed by the clinicians to feed the critically ill children.

**Conclusions:**

Regular in-service training of clinicians on feeding critically ill children, provision of logistics and specialized personnel in the ICU are recommended to reduce possible infant and child mortality resulting from suboptimal feeding.

## Background

Globally, less than 2 % of children admitted into hospitals require intensive care services [1]. However, children admitted to neonatal and paediatric intensive care units (ICUs) are at increased risk of mortality due to the complex nature of their illnesses posing a burden on paediatric care [1–4]. Evidence suggests that 50 % of critically ill children admitted to the intensive care are prone to malnutrition due to increased metabolic rate from their illnesses [2, 5]. It is important for the critically ill to receive constant optimal feeding to facilitate recovery especially with breastmilk since it is rich in immune components and nutrients [6–9]. Once a baby is born there are three threats to his/her survival including temperature of the environment, infections and most significantly feeding [10]. Feeding at any stage of life is a basic need for survival [11]. The newborn faces the reality of life to start feeding within half an hour from birth to transition to adopt to the extra uterine life in order to meet the physiological needs of the body [8, 9, 11].

Several determinants affect the feeding of the critically ill child. For instance, a seriously ill child may have an increased demand for nutritional needs which requires accurate and swift interventions [12]. Often the children are admitted into the ICUs on accounts of conditions such as neonatal jaundice, preterm, birth asphyxia, respiratory distress syndrome, congenital abnormalities, poisoning, juvenile diabetes mellitus and sickle cell crisis [1, 3, 4]. These critical illnesses cause impaired metabolism in the child [12, 13]. Maternal age and prematurity also affect the feeding in the intensive care units [14] as the reflexes of preterm babies impacts feeding [15]. Clinicians in the ICU usually measure gastric residual volume to determine the tolerance level of a critically ill child before commencing enteral feeding [16]. Overall, the feeding of critically ill children in the ICU is largely dependent on experts’ opinion and clinical experiences [11].

Parenteral feeding using pharmaconutrients is one major means of feeding the critically ill child in the ICU where an intravenous access is created for the essentially refined nutrients to be delivered in well estimated quantities [17]. Mostly, enteral feeding is the recommended feeding method for critically ill children in the ICU [18, 19] which differs from the usual breastfeeding and may cause feeding intolerance and decreased nutritional outcomes [2, 13, 20, 21]. Enteral feeding in itself may cause underfeeding and therefore need to be complemented with volume-based feeding [11]. However, human milk still remains the main nourishment for children, therefore, mothers breastfeed and or express milk for feeding of their critically ill children in the ICU [7, 14]. Experts in nutrition and dietetics are sometimes required to estimate the number of micronutrients and mix-feeds for critically ill children in intensive care [22]. The feeds are calculated based on the weights of the children to meet the nutritional demands of the body to support early recovery [6, 23]. In the NICU for example, Kangaroo Care has been used to reduce hypothermia which encourages infants to feed well [24].

The population of children requiring ICU services are increasing [11]. The clinicians conduct the feeding of these critically ill neonates though the number of patients outweigh the personnel. For example, the average annual admissions to NICUs across Ghana is about 3000 children [25]. These newborns start their life in the intensive care due to illnesses and or events superimposed on them in the course of pregnancy, labour and the perinatal period [8]. This interrupts their feeding patterns and recovery rate. Yet, ICU services in Ghana are not accessible to the masses and mainly exist at regional facilities. Existing feeding protocols of critically ill infants and children in the ICU [18, 19] may not be directly applicable in the Ghanaian context which affect feeding of older children. This is because several nuances exist to individualized nutritional support for the heterogenous population of infants and children in ICUs [26]. There is therefore the need to explore and describe the specific feeding processes and the determinants of critically ill children from experts’ perspectives. The primary research question of this study was, what are the feeding processes and the determinants for feeding the critically ill child in Ghanaian NICUs and PICUs? Hence this study adopted an exploratory descriptive design on the basis of gathering varied contextual perspectives of the participants via interviews and focus group discussions (FGDs) to answer the research question.

## Methodology

### Aim and design

This current study adopted a descriptive qualitative exploratory design aimed at exploring and describing the determinants of feeding and the feeding processes of critically ill children in the intensive care units. This design was the most appropriate for this qualitative study [27–29]. This is because it allowed the researchers to gather rich data on feeding the critically ill child from a realistic perspective in a natural setting [29, 30]. A qualitative approach is usually appropriate in a study where there are fairly new or little research conducted in an area within a particular context which in this case is feeding critically ill infants in the Ghanaian context [27, 28]. In the current study, both interviews and FGDs strategies were adopted due to the exploratory nature of the study and also to obtain thick data from varied perspectives of the participants to constructively described the feeding of a critically ill child in Ghanaian ICUs.

### Setting

The study was conducted in two public tertiary teaching hospitals in Ghana. The Korle-bu Teaching Hospital (KBTH) is the premier teaching hospital of Ghana which serves major parts of Southern Ghana and neighboring countries like Togo and Nigeria. The Tamale Teaching Hospital (TTH) is the only public tertiary teaching hospital in the five regions of Northern Ghana. These facilities were selected because they are the main referral centers for intensive care services of critically ill children in Ghana. The clientele of the selected hospitals extends beyond the inhabitants of those regions to include patients from the middle parts of Ghana and neighboring countries like Burkina Faso, Northern Togo, and the Ivory Coast. Therefore, the selection of these two facilities was representative enough to gather the information about the phenomenon of feeding these babies in the Ghanaian context. The age of patients in Ghanaian NICU and PICU ranges from birth to 12 years. In Ghana, some neonates and infants are managed at a subunit in the PICU due to increasing patients’ load and logistics reasons.

### Sampling and data collection procedures

The inclusion criteria of participants were clinicians who were nurses, midwives and physicians working at the neonatal and paediatric intensive care units for at least 2 years. Clinicians with less than 2 years working experiences in these ICUs as well as those outside the ICU in the setting were excluded. They were purposively recruited on voluntary basis based on the inclusion criteria. Ethical clearance was sought, and permission was obtained from the department of child health. A list of clinical staffs (nurses, midwives, and physicians) who worked at the neonatal and paediatric intensive care units for at least 2 years were obtained from the department administrator and with the help of the unit heads, those staff who were interested were recruited into the study. A total of 12 individual interviews and 6 focus group discussions (FGDs) were conducted (*N* = 42). These participants were purposely recruited for the study because they directly handled the feeding processes and care of the critically ill children and thus could better provide a description of the feeding process. An interview guide was developed based on the aim of the study. Key questions included: *how do you feed the critically ill child in the intensive care*? ; *Can you please describe the specific feeding processes of critically ill children? And what are the determinants of feeding a critically ill child at the intensive care?* The guide was pre-tested, and findings were used to refine the questions. Participants were informed that participation was voluntary. All interviews were conducted in English.

The interviews and the FGDs were conducted at the staffs’ rest rooms to minimize interferences and get quality voice recording and to ensure privacy. Information sheet, consent form as well as consent for voice recording were obtained from participants who volunteered to partake in the study. Initially, FGDs were conducted followed by individual one on one experts’ interviews. The individual interviews took about 30 min and FGDs lasted for at least 1 h. Data saturation was reached where no new information was obtained. Data was audiotaped and transcribed verbatim.

### Data analysis

The qualitative data was content analyzed [29, 30, 31, 32] after it was transcribed verbatim from audio to text. Data transcription and analysis were done concurrently to ensure that, emerging themes and sub-themes were further probed in subsequent interviews. Field notes were typed and compared to interview and FGD scripts to ensure data triangulation. In the works of Braun and Clarke (2006), codes are labels recognizing what is of attention in the data while themes are commonly occurring patterns across a data set gathered around a dominant organizing concept [29]. The transcripts were read several times by all the researchers and initial coding done manually by assigning names and phrases to sentences that depicted the meanings of those sentences. The researchers then used MAXQDA Plus version 2020 qualitative software to further code and developed sub-themes using descriptive and inductive coding techniques which aggregated into major themes which were: determinants of feeding and feeding processes. Sub-themes were then grouped under these two main themes to obtain the results. The researchers later met to agree on the themes and to ensure that, participants’ perspectives were kept intact.

### Trustworthiness of the study

In this study, some of the participants were randomly contacted to clarify their statements in order to ensure member checking. The same interview guide was used to interview all participants. Concurrent data collection and analysis ensured that, emerging themes were further probed in subsequent interviews. The verbatim transcripts from participants were used to back participants perspectives in order to keep their opinions intact. The field notes taken during data collection were used to verify the audio data. The researchers met to build a consensus on the findings of the study and arrived at the conclusion that the findings were representative of participants’ opinions.

### Ethical considerations

All methods were carried out in accordance with all guidelines and regulations under ethics approval and consent to participate. Ethical clearance was obtained from the Institutional Review Boards of the two hospitals (TTH/R&D/127, and STC/IRB/000145/2019). Information sheets and consent forms were provided to participants for them to voluntarily consent to participate in the study. Alpha-numeric codes were used to represent participants for the sake of anonymity. Data cleaning was done before data analysis in order to remove all identifiable data. Participants were informed that voluntary participation meant they could withdraw from the study at any stage without any consequences.

## Results

### Participants’ background

The participants were between the ages of 26–52 years with an average age of about 38 years. They included paediatric nurses, paediatricians, neonatal nurses, registered nurses, and midwives. The staff had varied ethnic backgrounds and religious backgrounds. The staff had an average working experience of about 15 years and a maximum working experience of 26 years. About 10(33 %) of the participants recruited for the FGDs were specialized in neonatal nursing, paediatric nursing, critical care, and paediatrics. Overall, 16(38 %) were specialists and this was necessary to enrich the study findings with experts’ inputs and 75 % of all the participants were females. Refer to Table 1 for details of the participants demographics.

**Table 1:** Participants’ demographics (*N* = 42)

Background information of FGDs participants (*n* = 30)				
**Code**	**Number (characteristics)**	**Age range (years)**	**SETTING (ZONE)**	**Years of working experience**
TSD1	5 (1-RN, 4-Neonatal Nurses)	30-43	TTH (Northern)	5-21
TSD2	5 (All medical doctors)	25-28	TTH (Northern)	2-3
TSD3	5 (2-PN, 3-RN)	28-36	TTH (Northern)	4-11
KSD1	5 (2-RN, 1-CCN, 1-PN, RNA)	26-32	KBTH (Southern)	5-8
KSD2	5 (2-RNA, 1-RN, 2-CCN)	29-35	KBTH (Southern)	4-10
KSD3	5 (1-RNA, 3- RN, 1- DP)	28-32	KBTH (Southern)	5-7
Background information of individual interview participants (*n* = 12)				
**Code**	**Sex**	**Age (years)**	**Profession/Speciality**	**Years of working experience**
TSI1	F	32	General practitioner	5
TSI2	F	33	Neonatal Nurse	9
TSI3	F	36	Neonatal Nurse	21
TSI4	F	41	Nurse Practitioner (DDNS)	19
TSI5	M	36	Registered Nurse (PNO)	13
TSI6	M	42	Consultant paediatrician	15
KSI1	F	43	Specialist paediatrician	16
KSI2	M	40	Resident paediatrician	15
KSI3	F	30	Post-Basic Midwife	8
KSI4	F	26	Registered Nurse	6
KSI5	F	51	Nurse Paediatrician (DDNS)	26
KSI6	F	52	Midwife (DDNS)	24

Key: *RN* Registered Nurse, *CCN* Critical Care Nurse, *PN* Paediatric Nurse, *RNA* Registered Nurse Assistant, *DP* Doctor Paediatrician, *T* Tamale, *K* Korle-bu, *S* Staff, *I* Interview, *D*= Focus Group Discussion

### Themes and sub-themes

Two major themes emerged after the inductive content analysis; determinants of feeding and feeding processes of the critically ill child in intensive care. The determinants of feeding had three sub-themes evolving from the findings: experts’ knowledge/skills, medical conditions, and gestational age. The feeding process in intensive care had four sub-themes: parenteral, enteral, cup feeding and breastfeeding.

### Determinants of feeding

#### Experts’ knowledge/skills

It emerged from the study that knowledge and skills of clinicians at the intensive care units were the major determining factors to feed the critically ill child. This is illustrated in the statement below in the excerpts of a pediatric nurse:

*“Here, we do collaborative care where we involve other specialists like the nutritionists and dieticians, eye specialists, physiotherapists and other team members to share ideas and skills on how to go about the feeding. There is the need for specialized skills and knowledge in order to reduce the risk of aspiration.”****KSI5***.

Some reported that, due to inadequate knowledge and skill, some clinicians will not feed the babies if they did not feel confident and skillful for fear of aspiration and death as recounted by a neonatal nurse:

*“For feeding in the intensive care unit, there is the need for requisite knowledge and being careful in order to avoid aspiration. So, for some of the staff, in order not to expose themselves, they leave the breast milk untouched without attempting to feed the babies as several instances have occurred on this ward where feeding of the babies led to deaths. This is because aspiration in preterm babies is very dangerous and most staff too working here have not specialized in intensive care and care of neonates”****TSD1A***.

#### Gestational age

The gestational age of the baby was mentioned as one of the determinants for feeding as it directly influences the reflexes and ability of the baby to suckle. This was summarized by two participants as:

*“In feeding the newborn babies, one factor to consider is the gestational age of the baby as this goes along with the maturation and the presence of certain reflexes in the baby i.e. ability to suckle and ability to swallow”****TSD1C***.

*“Then we have the small preterm whose gestational ages are so low to an extent that, their sucking and swallowing reflexes are not well established making it difficult for them to feed. That determines the feeding choice. In such cases, we try and avoid the oral feeding method.”****KSI1***.

#### Child’s medical conditions

From the participants perspectives, one major determinant of feeding the critically ill child is the medical condition of the child. A paediatrician and neonatal nurse described this in the extracts below:

*“Per observations, it is not entirely the fault of the staff as the medical conditions of certain babies make some staff afraid to feed them for fear of choking or causing aspiration which can cause death and in other cases of congenital anomaly feeding can facilitate the death of the baby”****TSD1D***.

*“We have babies who definitely cannot be fed orally due to their medical conditions. Therefore, their medical conditions determine their feeding method. So, we do not want to feed critically ill babies because of the risk of aspiration,”****KSI1***.

Some of the participants recounted infections, preterm, and some congenital abnormalities like cleft lip and cleft palate hinder the feeding:

*“Stable babies are being fed well by staff but due to the fear of baby dying as a result of aspiration, preterm babies, critically ill babies, and babies with congenital anomalies are not fed well”****TSD1C***.

### Feeding process in ICU

#### Parenteral feeding

A nurse manager who is a nurse paediatrician described how critically ill children are fed parenterally in the unit:

*“We calculate based on the maintenance, what is required for the child per weight. To start with we do not give full feed i.e. we do not give the 100 % feed, because they are now starting to feed. We start from 25 % of their daily weight, then fluids will be 75 %. Then when the child progresses, we proceed to 50 % feed to 50 % IV fluids. when the child recovers and gains full consciousness, we reverse the feeding to 75 % feeds and 25 % IV fluids. Then gradually we wean the child off the IV fluids and put the child on full feeds”****KSI5***.

#### Enteral feeding

Four of the participants summarised enteral feeding as one of the feeding processes of critically ill children in the intensive care as follows:

*“For the feeding because they are critically ill, we normally start with the enteral feeding through NG tube. We calculate the drugs, the feeds, and monitor the patients very well.”****KSI3***.

*“We use a nasogastric tube, then oral before we attach the baby to breast****” TSD3B***.

*“Not all mothers can express the milk, so we have to be giving them formula through an NG tube. So now sometimes we are doing mix-feeding”****KSI5***.

*“But again, you need to weigh the risks and the benefits. So, sometimes there may be respiratory distress but we may pass NG tubes and elevate the head end to feed the baby. Then we do the feeding and not leave the baby for the parents to feed to reduce the risk of aspiration****” KSI1***.

#### Cup/top-up feeding

The critically ill children in the NICU are often fed by cup and spoon as they are unable to attach to the breast for breastfeeding. Extracts from the experts are presented below:

*“So, it is a big cup and a small cup, two cups so one of them has the measurement on it so after breastfeeding you top up over an amount so that’s the way we do it. After that we allow the mother to express the milk then we use a small spoon to feed so when they are going home, we sell the feeding containers to them. The feeding container set consist of jar for milk storage, cup, and spoon for feeding****” KSI6***.

*“For those babies who cannot attach to the breast, we encourage the mothers to express the breast milk. The nurses may either feed by NG tube or cup and spoon. We encourage the mothers to put to breast but then also express so that the mother herself does the cup and spoon feeding of the baby in preparation for going home”****KSI1***.

*“We teach them how to do cup and spoon; after breastfeeding, you top up with breast milk so that the baby will gain weight”****KSD1C***.

#### Breastfeeding

Participants indicated that, breastfeeding is employed as a feeding process when the child’s condition is stable and can suck the breast milk:

*“For feeding the critically ill, mothers come into the NICU every three hours. When they come for those babies who can attached to the breast and feed, we ask the mothers to breastfeed them. For those babies who are feeding but cannot attached to the breast for feeding we encouraged the mothers to express the breast milk”****KSI1***.

*“Sometimes the mothers think that once you just put baby to breast and baby sucks then the baby has had breast milk. There are instances where the mother is not lactating in other words the mother is not producing breast milk and so you would see baby put to breast, but baby did not really get breast milk. In those instances, we advise them that after sucking they should try and express and give it as well since you cannot quantify the amount they have sucked but you can quantify the amount expressed to be given as top up to the baby”****TSI2B***.

Breastfeeding in the intensive care comes with some challenges including lack of storage for expressed breast milk and poor lactation by some mothers:

*“The challenge too is that some mothers can produce more milk than what the baby needs, but we do not have the storage facility for the expressed milk. Normally, expressed milk can only be used for a maximum of 4 hours. So, the absence of the storage facility for the milk in the NICU also adds to the stress of the mothers because they go through a lot of pains to produce the milk”****TSD1E***.

Breastfeeding is done 2 hourly and for babies like preterm who are unable to suck themselves, the experts use alternative method like cup feeding or enteral feeding with expressed breast milk.

*“Also, you talk of feeding too where you must explain to mothers and encourage them to breastfeed mostly two hourly and after that express the milk.”****TSD1A***.

## Discussions

Experts’ knowledge, child’s medical condition, and the gestational age were the key determining factors for feeding critically ill children in the intensive care units. The expert’s skills and understanding of the child’s condition was what determined the quantity to feed as well as the feeding method adopted at both NICU and PICU. These findings are consistent with the results of a systematic review on nutritional support for critically ill children [12]. With regards to child’s medical condition, most of the participants were hesitant to feed the very critically ill child because of fear of causing aspiration. Conceivably, preterm babies and terminally ill unconscious older children have poor reflexes and are unable to feed well and this made the experts avoid feeding the critically ill children for fear of aspirations and deaths. This finding was contrary to the recommendations of the United Nations International Children’s Emergency Fund (UNICEF) and World Health Organisation (WHO) on meeting the nutritional needs of a child especially during illness; in their joint publication in the Lancet, they identified infrequent feeding as one of the three events that can threaten the survival of children [10]. The participants’ avoidance of feeding in this current study due to fear of causing aspiration in the critical care units may be legitimate since aspiration due to feeding has been reported in a study conducted in Brazil to investigate the source of mortality in the paediatric intensive care unit (PICU) [4]. These stands of the staff may be attributable to the inadequate specialized clinicians in the ICU. These findings may be necessary to echo calls for more specialized training of doctors and nurses as well as providing continuous in-service training on the care of critically ill children especially on feeding in the NICU and PICU. This will refresh their knowledge and skills and avert any possible death of children in the ICUs relative to the effects of feeding. Experts in another study provided an account of how feeding resulted in aspiration and subsequent death of critically ill children [1]. Perhaps regular training of clinical staff on feeding of critically ill children in the NICUs and PICUs may ultimately address this avoidable phenomenon. This may go a long way to reduce neonatal, infant, and child mortality in Ghana.

The researchers found that participants fed the critically ill children mainly through a nasogastric tube using formula feeds, breast milk, fluids, and other refined feeds which is consistent with reports from other studies [2, 19]. One paediatrician lamented the effects of the enteral feeding method on children with tachypnoea and respiratory distress as it may cause intolerance and worsen the condition of the critically ill child in the ICU [2, 13, 20, 21]. Parenteral feeding was found in another study to be useful in most cases especially at the PICU to meet the nutritional needs of the critically ill child [17]. One feeding method adopted for feeding the critically ill that emerged from this Ghanaian qualitative study was the use of cup and spoon. Participants used cup and spoon to feed infants who were unable to latch onto the breasts for breastfeeding. They also conducted top-up feeding with the same cup and spoon for children who were poorly breastfed to help them meet their nutritional needs. Perhaps, the different methods adopted by participants in feeding the critically ill children were due to the different conditions of the children admitted in the NICU and PICU. It also afforded the participants an opportunity to give bespoke care to them. Due to the bespoke nature of care that has to be given to children at the ICUs, it is recommended that, all the resources; both human and material (refrigerator for infant feeds, and feed banks) should be provided to make their work easier.

### Limitations

The limitation of this study was that the sample was small, and the participants were mostly interviewed after their shift which may have confounded the results due to fatigue. Future research may scale up the sample to include more facilities to develop a comprehensive feeding criterion of critically ill patients within the context of the Ghanaian setting.

## Conclusions

The study found that feeding of critically ill children in the intensive care is an activity which most health professionals avoid due to fear of causing aspiration which can lead to death. The main feeding process adopted were breastfeeding, parenteral feeding, enteral feeding and cup and spoon feeding. Regular in-service training of clinicians on feeding critically ill children, provision of logistics, and specialized personnel at such units are recommended to reduce the infant and child mortality resulting from suboptimal feeding. Future research may look at outcomes of neonates and children from NICU and PICU to promote the standardization of protocols including feeding practices in the Ghanaian setting.

## Data Availability

Qualitative data in form of transcripts and audios are available upon reasonable request from the research team.

## References

[1] Ibiebele I, Algert CS, Bowen JR, Roberts CL (2018). Pediatric admissions that include intensive care: a population-based study. BMC Health Serv Res.

[2] Haney A, Burritt E, Babbitt CJ (2018). The impact of early enteral nutrition on pediatric acute respiratory failure. Clin Nutr ESPEN.

[3] Rennick JE, Childerhose JE (2015). Redefining success in the PICU: New patient populations shift targets of care. Pediatrics.

[4] El Halal MGDS, Barbieri E, Filho R, Trotta E, Carvalho P (2012). Admission source and mortality in a pediatric intensive care unit. Indian J Crit Care Med.

[5] Stapleton RD, Jones N, Heyland DK (2007). Feeding critically ill patients: what is the optimal amount of energy?. Crit Care Med.

[6] Larsen BMK, Beggs MR, Leong AY, Kang SH, Persad R, Garcia Guerra G (2018). Can energy intake alter clinical and hospital outcomes in PICU?. Clin Nutr Espen.

[7] Sorce LR, Curley MAQ, Kleinpell R, Swanson B, Meier PP (2020). Mother’s own milk feeding and severity of respiratory illness in acutely ill children: an integrative review. J Pediatr Nurs.

[8] Whetten CH (2016). Cue-Based Feeding in the NICU. Nurs Womens Health.

[9] Cuttini M, Croci I, Toome L, Rodrigues C, Wilson E, Bonet M (2019). Breastfeeding outcomes in European NICUs: impact of parental visiting policies. Arch Dis Child Fetal Neonatal Ed.

[10] Clark H, Coll-Seck AM, Banerjee A, Peterson S, Dalglish SL, Ameratunga S (2020). A future for the world’s children? A WHO–UNICEF–Lancet Commission. Lancet.

[11] McClave SA, Saad MA, Esterle M, Anderson M, Jotautas AE, Franklin GA (2015). Volume-Based Feeding in the Critically Ill Patient. J Parenter Enter Nutr.

[12] Joffe A, Anton N, Lequier L, Vandermeer B, Tjosvold L, Larsen B, et al. Nutritional support for critically ill children (Review) summary of findings for the main comparison. Cochrane Database Syst Rev. 2016. 10.1002/14651858.CD005144.pub3. 10.1002/14651858.CD005144.pub3 PMC6517095 27230550

[13] López-Herce J, Mencía S, Sánchez C, Santiago MJ, Bustinza A, Vigil D (2008). Postpyloric enteral nutrition in the critically ill child with shock: a prospective observational study. Nutr J.

[14] Casey L, Fucile S, Dow KE (2018). Determinants of successful direct breastfeeding at hospital discharge in high-risk premature infants. Breastfeed Med.

[15] Joseph RA, Evitts P, Bayley EW, Tulenko C (2017). Oral feeding outcome in infants with a tracheostomy. J Pediatr Nurs.

[16] Tume LN, Sciences A, Hill B, Uk BS (2020). Europe PMC funders group gastric residual volume measurement in UK paediatric intensive care units: a survey of practice. Pediatr Crit Care Med.

[17] Skillman HE, Mehta NM. Nutrition therapy in the critically ill child. Current Opinion in Critical Care. 2012;18(2):192-8. 10.1097/MCC.0b013e3283514ba7 22322263

[18] Jouancastay M, Guillot C, Machuron F, Duhamel A, Baudelet J-B, Leteurtre S (2021). Are nutritional guidelines followed in the pediatric intensive care unit?. Front Pediatr.

[19] Tume LN, Valla FV, Joosten K, Jotterand Chaparro C, Latten L, Marino LV (2020). Nutritional support for children during critical illness: European Society of Pediatric and Neonatal Intensive Care (ESPNIC) metabolism, endocrine and nutrition section position statement and clinical recommendations. Intens Care Med.

[20] Lee JH, Rogers E, Chor YK, Samransamruajkit R, Koh PL, Miqdady M (2016). Optimal nutrition therapy in paediatric critical care in the Asia-Pacific and Middle East: a consensus. Asia Pac J Clin Nutr.

[21] Tume LN, Valla FV (2018). A review of feeding intolerance in critically ill children. Eur J Pediatr.

[22] Fernández R, Urbano J, Carrillo Á, Vivanco A, Solana MJ, Rey C (2019). Comparison of the effect of three different protein content enteral diets on serum levels of proteins, nitrogen balance, and energy expenditure in critically ill infants: study protocol for a randomized controlled trial. Trials.

[23] Dao DT, Anez-Bustillos L, Cho BS, Li Z, Puder M, Gura KM (2017). Assessment of micronutrient status in critically ill children: challenges and opportunities. Nutrients.

[24] Justyna W (2017). A test of kangaroo care on preterm infant breastfeeding. Physiol Behav.

[25] Ministry of Health. The Republic of Ghana. www.mohgh.Health Sector Medium Term Development Plan 2014–2017. 2017.

[26] Mehta NM, Skillman HE, Irving SY, Coss‐Bu JA, Vermilyea S, Farrington EA, McKeever L, Hall AM, Goday PS, Braunschweig C. Guidelines for the provision and assessment of nutrition support therapy in the pediatric critically ill patient: Society of Critical Care Medicine and American Society for Parenteral and Enteral Nutrition. Journal of Parenteral and Enteral Nutrition. 2017;41(5):706-42. 10.1177/0148607117711387 28686844

[27] Acheampong AK, Naab F, Kwashie A. The voices that influence HIV-positive mothers’ breastfeeding practices in an urban, Ghanaian society. Journal of Human Lactation. 2018;34(1):176-83. 10.1177/0890334417740345 29268662

[28] Creswell JW. Qualitative, quantitative and mixed methods approaches. Sage; 2014.

[29] Belotto MJ (2018). Data analysis methods for qualitative research: managing the challenges of coding, interrater reliability, and thematic analysis. Qual Rep.

[30] Albert M, Mylopoulos M, Laberge S (2019). Examining grounded theory through the lens of rationalist epistemology. Adv Heal Sci Educ.

[31] Rieger KL (2019). Discriminating among grounded theory approaches. Nurs Inq.

[32] Braun V, Clarke V (2006). Using thematic analysis in psychology. Qual Res Psychol.

